# Changes in the Success and Characteristics of Tobacco Dependence Treatment before and during the COVID-19 Pandemic: Clinical Sample Comparisons

**DOI:** 10.3390/medicina60091459

**Published:** 2024-09-06

**Authors:** Lenka Stepankova, Kamila Zvolska, Alexandra Pankova, Jakub Rafl, Gleb Donin, Ales Tichopad, Eva Kralikova

**Affiliations:** 1 Centre for Tobacco Dependent of the 3rd Medical Department, Department of Endocrinology and Metabolism, First Faculty of Medicine, Charles University in Prague and General University Hospital, 12808 Prague, Czech Republic; lenka.stepankova@lf1.cuni.cz (L.S.); alexandra.pankova@lf1.cuni.cz (A.P.); 2 Institute of Hygiene and Epidemiology, First Faculty of Medicine, Charles University in Prague and General University Hospital, 12808 Prague, Czech Republic; 3 Department of Biomedical Technology, Faculty of Biomedical Engineering, Czech Technical University in Prague, 27201 Kladno, Czech Republic; rafl@fbmi.cvut.cz (J.R.); gleb.donin@fbmi.cvut.cz (G.D.);

**Keywords:** tobacco dependence, treatment, COVID-19 pandemic

## Abstract

*Background and Objectives*: There is little information on changes in the process and outcomes of intensive tobacco dependence treatment during the COVID-19 pandemic. The following characteristics were evaluated: interest in treatment, the number of face-to-face or telephone follow-ups, the duration of pharmacotherapy use, and the success rate. The aim of our study was to compare the number of patients who entered tobacco dependence treatment programmes and evaluate the one-year success rate in patients three years before and three years after the COVID-19 pandemic. *Materials and Methods*: A single-site retrospective cohort study using data from patients treated at the Centre for Tobacco Dependence in Prague, Czech Republic, between 2017 and 2022 (n = 2039) was performed. The one-year abstinence rate was validated by measuring carbon monoxide in exhaled air (6 ppm cut-off). Patients were divided into two groups: the group for which treatment was initiated in 2017–2019 (i.e., before the COVID-19 pandemic, BC; n= 1221) and the group for which treatment was initiated in 2020–2022 (i.e., during the COVID-19 pandemic, DC; n = 818). *Results*: No significant differences in the success rate of tobacco dependence treatment were found between the two groups (BC group, 40.5% (494/1221) vs. DC group, 42.2% (345/818)) (*χ*^2^ (1, *N* = 2.039) = 0.6, *p* = 0.440). Furthermore, differences were not found in sex, education level, age at first cigarette, the duration of pharmacotherapy use, or the number of in-person visits. In contrast, there was an increase in the number of telephone contacts between the groups (18.7% (SD = 17.5%) vs. 32.9% (SD = 18.2%), *p* < 0.001). *Conclusions*: The number of patients who started treatment during the COVID-19 pandemic decreased by one-third compared to that during the 3-year period before the pandemic. The overall treatment success rate did not change significantly even with the increase in the number of telephone visits with the therapist.

## 1. Introduction

The success of tobacco dependence treatment depends on many factors, including the patient, the therapist, and the treatment approach. The type and duration of pharmacotherapy (first-line evidence-based drugs, including nicotine replacement therapy, varenicline, bupropion, and cytisinicline) and the number of therapeutic contacts are repeatedly cited as the most important factors of intensive treatment success [1,2,3]. Identifying tobacco users, recommending cessation of smoking or other forms of tobacco use, and offering adequate treatment should be part of every clinical contact with a smoker [4]. There are limited data on how smoking behaviour and the availability and success of intensive tobacco dependence treatment were affected by the COVID-19 pandemic, and virtually none of these studies have been conducted in the Czech Republic.

The COVID-19 pandemic led to a significant increase in stress levels for the majority of the population and made it impossible to travel, meet with friends, and even leave the house or go to work, especially at the height of the pandemic. This was understandably reflected in a change in behavioural patterns and therefore in the number of cigarettes smoked, as confirmed in a study by Dutch authors describing a synergistic and dose–response relationship between stress levels and smoking during the COVID-19 pandemic. A multinominal logistic regression analysis revealed that smokers who were under moderate stress either increased (OR = 2.37; 95% CI: 1.49–3.78) or reduced (OR = 1.80; 95% CI: 1.07–3.05) their smoking frequency. Severely stressed smokers were even more likely to have either an increased (OR = 3.75; 95% CI: 1.84–7.64) or reduced (OR = 3.97; 95% CI: 1.70–9.28) smoking frequency [5]. The change in smoking behaviour, i.e., an increase in the average number of cigarettes smoked per day during the pandemic for some respondents, and a decrease for others, was also confirmed independently by studies in Italy [6], the US [7], Germany [8] and Poland [9]. Increased nicotine use in the first month of lockdown was reported in a study conducted in Greece [10]. In a broader review article, Mellos and Paparrigopoulos reported that substance use during the COVID-19 pandemic in Greece and other countries varied widely according to drug availability and other factors; additionally, they reported that the relationship between addiction and the COVID-19 pandemic was not direct and that untimely generalisations should be avoided [11]. In the nationwide representative survey in the UK, among current smokers (n = 329), one-quarter (25.2%, n = 86) reported smoking more than usual, 50.9% (n = 174) reported smoking the same amount, and 20.2% (n = 69) reported smoking less. Significant associations were observed between different smoking behaviour groups and psychosocial factors; deterioration of mental health and psychosocial well-being were linked to increased smoking [12]. We have a minimal amount of information about changes in the behaviour of smokers in the Czech Republic during the pandemic. The only source states a slight decrease in smoking prevalence from 24.9% in 2019 to 23.1% in 2020 [13].

The COVID-19 pandemic led to less access to (and thus fewer) nonurgent face-to-face appointments with health professionals and therapists. This situation also affected the field of addiction treatment, as reflected in the vast majority of available sources [11,14,15,16]. An exception is a study conducted in the UK that did not report a decline in the number of face-to-face visits at smoking cessation centres according to the Office for National Statistics [17]. Many health professionals in general, including those specialising in tobacco dependence treatment, have been looking for new ways to deliver treatment. As a result, there has been a significant increase in the provision of telephone consultations, video consultations, chat applications, and other remote forms of treatment [18,19,20,21].

Reports on tobacco dependence treatment outcomes during the pandemic and the impact of the pandemic on modifiers of success are mixed. Different papers report different significant changes in interest in quitting, different effects on treatment success, and other characteristics after the pandemic compared to the prepandemic period. The study by Jackson et al. revealed not only increased use of smoking cessation services during the COVID-19 pandemic and increased use of medications for tobacco dependence, specifically varenicline [22], but also a higher rate of smoking cessation (21.3 versus 13.9%, ORadj = 2.01, 95% CI = 1.22–3.33) in the UK [17]. A slight increase in the success rate of tobacco dependence treatment was also reported in one other source [23]. However, other studies did not have such optimistic results [24,25,26]; Turan et al. reported a reduction in the success rate of tobacco dependence treatment in their outpatient facility, citing increased stress levels, depressive symptoms, premature discontinuation of cessation medication, and coronavirus infection as the main negative influences [25]. Other studies did not report a significant change in success rates or treatment outcomes.

Data from the Czech Republic on tobacco dependence treatment during the COVID-19 pandemic have not yet been published.

The aim of our retrospective study was to compare the number of patients who entered tobacco dependence treatment programmes and evaluate the one-year success rate in patients at the Centre for Tobacco Dependence Treatment three years before and three years after the COVID-19 pandemic. Another objective was to evaluate changes in the proportion of patients with telephone contact with a therapist, the duration of pharmacotherapy, and patient demographic characteristics.

## 2. Materials and Methods

The analyzed dataset included 2141 patients from the centre who started their treatment between 2017 and 2022, completed it before 2024, and had at least one personal visit to the centre. These patients were divided into two groups: the before COVID-19 (BC) group, whose treatment started in 2017–2019 and ended no later than 2020, and the during COVID-19 (DC) group, whose treatment started in 2020–2022 and ended no later than 2023. For consistency, patients who started treatment in 2017–2019 but did not complete treatment by the end of 2020, for various reasons, were excluded, as were those who did not undergo carbon monoxide (CO) measurement one-year postcessation, resulting in a final sample size of 2039. An intention-to-treat approach was applied; all treatment-seeking patients smoking tobacco were included, while exclusive users of electronic cigarettes were excluded. Treatment success was evaluated using the Russell Standard [27]. One-year self-reported abstinence from all forms of tobacco was assessed, validated by the measurement of CO in exhaled air, measured with a Micro + ™ Smokerlyzer^®^ (Bedfont^®^ Scientific, Kent, United Kingdom) with a cut-off of 6 ppm.

### 2.1. Description of Treatment

All enrolled patients had at least one baseline screening visit, during which each patient received detailed information and signed a consent form, the patient’s medical and smoking history was taken, and self-help materials and leaflets were provided to the patient. The majority of patients (90.6% overall) then underwent a psychobehavioural intervention provided by a physician targeted at treating their tobacco dependence.

Data were routinely collected as part of the standard outpatient procedure. The treatment of patients at the Centre for Tobacco Dependence (General University Hospital outpatient clinic, Prague, Czech Republic) is conducted according to evidence-based treatment standards valid in the Czech Republic and worldwide and is individualised according to patient needs [1,28].

During the baseline visit, the individual’s degree of physical tobacco dependency was evaluated, a detailed medical history was collected, and selected psychodiagnostic tests and basic physical examinations were performed. During the psychobehavioural intervention at the second visit, physical and psychosocial considerations related to the patient’s tobacco dependence were discussed, such as the patient’s habits and rituals associated with smoking, and alternative suggestions were made with respect to both their tobacco use and to common situations and triggers that had led them to smoke previously. Additionally, pharmacotherapy options were introduced. The patient and therapist collectively decided upon an appropriate course of treatment, including the type and dose of pharmacotherapy (varenicline, bupropion, nicotine replacement therapy, cytisinicline, combination therapy, or none). Note: cytisinicline is an alkaloid isolated from various plant sources, varenicline is developed synthetically, both are nicotinic acetylcholine receptor partial agonists, and cytisinicline has a shorter half-life [29]. Finally, the target quit date and the date of the next follow-up visit were determined. A detailed description of the intervention is available at http://www.slzt.cz/intervention-structure (accessed on 1 May 2024).

Follow-up visits lasted 30 min on average. The first visit was planned within approximately 2 weeks of the target quit date, with the following visits taking place at approximately 2–4-week intervals until the third month of treatment; the frequency of further visits depended on the patient’s needs. The final follow-up visit was at 12 months after the quit date, at which anthropometrics, all changes related to medical history, and the total duration of pharmacotherapy use were recorded. The patients’ expired carbon monoxide (CO) levels were measured at every visit. Patients who did not successfully complete the first treatment cycle at our centre could enter a new cycle of treatment at least one year after the original planned quit day [30].

The authors assert that all procedures contributing to this work comply with the ethical standards of the relevant national and institutional committees on human experimentation and with the Helsinki Declaration of 1975, as revised in 2008. The study was performed with the consent of the Ethical Committee of General University Hospital 1798/12 S-IV and EK/VFN/18080 n. 798/18.

### 2.2. Data Analysis

The distribution of continuous data was assessed by visual inspection (histogram and normal probability plot) and by the Shapiro–Wilk test for normality. When the data obviously deviated from normal distribution, which was the case, a nonparametric Wilcoxon rank-sum test was used to compare the BC and DC groups. Otherwise, the independent two-sample *t*-test (for equal variance) was used. For categorical data, Pearson’s chi-squared test was used to compare the BC and DC groups. Data in tables are reported as mean (standard deviation) when the parametric test was used and as median [IQR] when the nonparametric test was used. Statistical analysis was performed in R (v4.3.2; R Core Team 2023), and *p* < 0.05 was considered to indicate statistical significance.

## 3. Results

### Cohort Description

The final analysis set of 2039 patients included 1221 patients in the before COVID-19 group and 818 patients in the during COVID-19 group. The number of patients entering treatment in the centre fluctuated between 2017 and 2022, as shown in Table 1. Overall, 33% fewer patients entered treatment during the COVID-19 pandemic period (2020–2022) than during the same-length period (2017–2019) prior to the pandemic. The majority of patients who sought treatment also underwent intensive therapeutic intervention (90.6% in total), with an increasing trend over the years (Table 1).

The demographic characteristics of the patients at admission are shown in Table 2. Some parameters did not change significantly between the periods, including the proportion of women, education level, and age at first cigarette. However, there were small but statistically significant changes in some parameters during the pandemic period. These included a slight increase in the age of patients starting treatment (45 vs. 43 years), a decrease in the number of cigarettes smoked per day (17 vs. 20 cigarettes), an increase in the number of years of regular smoking (27 vs. 24 years), and an increase in the median nicotine dependence score, as expressed by the FTCD (6 vs. 5 points) (Table 2).

The main parameter investigated was the change in the success rate of tobacco dependence treatment in patients with at least one visit for treatment at our centre and a follow-up visit after 12 months. There was a slight increase in treatment success in the DC vs. BC group, although the change in the abstinence rate did not reach statistical significance (42.2% vs. 40.5%, χ^2^ (1, n = 2.039) = 0.6, *p* = 0.440, Table 3).

The median number of personal visits per patient did not change significantly during the COVID-19 period (median 4 visits, mean 4.4 vs. 4.6), but there was an increase in the number of telephone contacts (median 2 vs. 1, mean 2.3 vs. 0.8), as shown in Table 4. Thus, the average number of telephone visits increased from 18.7% (SD = 17.5%) before the COVID-19 pandemic to 32.9% (SD = 18.2%) during the COVID-19 pandemic (*p* < 0.001; see Table 4). The proportion of patients who had an appointment and subsequently failed to attend on at least one occasion increased from 17% to 24% (*p* < 0.001).

The median duration of pharmacotherapy use was 53 days (mean 95 days) and did not differ significantly between the groups (see Table 5). During the COVID-19 period, varenicline imports stopped and cytisinicline became widely available, although these changes were not due to the pandemic. Thus, most of our patients who were previously taking varenicline gradually switched to cytisinicline, which continued to be used by most patients newly starting treatment during the COVID-19 pandemic.

## 4. Discussion

During the COVID-19 pandemic, the number of patients in our centre decreased by 33% compared to that before the pandemic (BC group versus DC group: 1221 vs. 818 patients). Although we anticipated lower availability of face-to-face visits, people were generally worried about visiting health facilities, so we started to offer remote telephone follow-ups and we contacted patients by telephone in cases of missed visits. To our surprise, the number of in-person visits per patient did not decrease significantly during the COVID-19 pandemic (median 4 visits per patient during the follow-up year); additionally, during the pandemic, the number of telephone visits increased (median 2 vs. 1 in the DC vs. BC group), which led to an increase in the total number of therapeutic contacts compared to before the pandemic. During the first stage of the COVID-19 pandemic, higher symptoms of anxiety and depression among Czech citizens were associated with behavioural, cognitive, and emotional changes [31]. We can assume that for some smokers, their increased anxiety about COVID-19 infection led to a reduced willingness to seek treatment for tobacco addiction. However, for smokers who sought treatment, it did not lead to a reduced number of visits.

Furthermore, it is important to emphasise the existing intercultural differences in the level of psychological difficulties caused by the COVID-19 pandemic and associated behavioural changes. In different countries or ethnic groups, the level of fear of infection and related anxiety and depression were of varying importance. According to the available sources, these were high in Germany [32] and Bangladesh [33] and moderate or low in Japan, the Czech Republic, Romania, and Cuba [34,35,36,37], if measured by the Fear of COVID-19 Scale (FCV-19S). Furthermore, psychological distress caused by COVID-19 can vary according to age, gender, and other parameters, which, however, cannot be fully investigated in all their complexity.

The second most important finding was that the COVID-19 pandemic did not significantly affect tobacco treatment success rates. The slight differences, with better treatment outcomes in the DC group than in the BC group (42% vs. 40%), may be explained by the increased number of telephone visits, while the total duration of pharmacotherapy use remained unchanged.

In addition, some studies showed similar effectiveness of remote and face-to-face visits, even if they were video-linked [38]. However, the type of remote contact may not play a role; some work has revealed no difference in the effectiveness of video calls and telephone contacts [39]. Overall, successful smoking cessation following treatment mostly decreased during the pandemic, and the possibility that virtual care is less effective than in-person treatment should be further explored [24]. In some countries, the number of patients seeking treatment for tobacco dependence declined significantly during the pandemic, e.g., in Canada [40]. The transition to remote care appears to have reduced the effects of socioeconomic and health barriers to treatment [41]. We can also consider other factors that could have influenced the slightly higher success rate during the COVID-19 pandemic, such as financial and technical difficulties associated with the pandemic, which could have affected the ability of smokers to buy cigarettes. Another factor may have been less time spent with their smoking friends.

Currently published data show that the impact of the COVID-19 pandemic on tobacco dependence treatment outcomes varies widely. The different sources listed above reported reduced or increased interest in treatment in some countries [11,14,15,16,17,40,41], higher [22,23] or lower [24,25,26] treatment success rates, and different effects on the duration of pharmacotherapy use. Consistent with most of these studies [11,14,15,16,41,42], our results showed less interest in entering treatment during the COVID-19 epidemic. We explain this decrease in the number of new patients during the pandemic by their fear of all interpersonal contact, especially in the risky environment of healthcare facilities.

They also showed a slightly higher success rate for those who did enter treatment, although not statistically significant, with an increased number of telephone contacts. Our results are in line with those of almost all studies, which reported that the COVID-19 pandemic increased the availability of remote visits [18,19,20,21,43], whether through video, chat, or telephone contact. However, the impact of the shift to remote types of services on the overall success of tobacco dependence treatment is not clear, and further studies, preferably using multivariate regression analysis, should be conducted.

### Strengths and Limitations of the Study

This study has several strengths, including the relatively large sample size of patients with repeated controls, the consistency of treatment over the two time periods, and the treatment and its detailed evaluation conducted by the same group of therapists over time.

Conversely, the study design (retrospective comparison of the period before and during the COVID-19 epidemic) did not allow for randomization of patients, which could lead to the occurrence of confounding factors that could not be controlled or measured. In addition, our results are based on data analysis from a single centre and cannot be automatically generalised to the entire population of the Czech Republic or other populations. Nevertheless, our centre is the largest in the country, providing more than 30% of all interventions for tobacco addiction carried out in the Czech Republic (both before and during the epidemic) [44], and the treatment was provided using a standard evidence-based approach following established international guidelines for the treatment of tobacco addiction. Therefore, we believe that our findings are relevant to other clinical sites and regions.

## 5. Conclusions

During the COVID-19 pandemic, the number of patients who started treatment at our outpatient clinic decreased by 33% compared to before the pandemic. The average number of personal visits did not change during the COVID-19 pandemic, and the number of telephone contacts significantly increased (*p* < 0.001). The increased total number of therapeutic contacts may have been reflected in the slight increase in treatment success (abstinence rate 42% vs. 40%) during the COVID-19 pandemic compared to before the pandemic, although this change in the success rate was not statistically significant (*p* = 0.440).

## Figures and Tables

**Table 1:** Number of new patients who completed psychobehavioural interventions by year.

Year	New Patients ^1^	Intervention ^2^
2017	555	457 (82%)
2018	368	312 (85%)
2019	298	281 (94%)
2020	278	265 (95%)
2021	237	234 (99%)
2022	303	299 (99%)

^1^ n; ^2^ n (%).

**Table 2:** Basic characteristics of the sample from 2017 to 2022.

	Total n = 2039 ^1^	Treatment Era		*p* Value
		Before COVID-19 n = 1221 ^1^	During COVID-19 n = 818 ^1^	
Sex				0.272 ^2^
Female	890 (44%)	545 (45%)	345 (42%)	
Male	1149 (56%)	676 (55%)	473 (58%)	
Age (years)	44 (13)	43 (13)	45 (13)	0.006 ^3^
Education level				0.323 ^2^
Basic	121 (5.9%)	68 (5.6%)	53 (6.5%)	
Apprentice	199 (9.8%)	109 (8.9%)	90 (11%)	
Secondary	1091 (54%)	666 (55%)	425 (52%)	
University	628 (31%)	378 (31%)	250 (31%)	
Number of cigarettes per day	19 (14)	20 (16)	17 (12)	<0.001 ^3^
Age at first cigarette (years)	15 (4)	15 (4)	15 (4)	0.152 ^3^
Duration of regular smoking (years)	25 (13)	24 (13)	27 (13)	<0.001 ^3^
FTCD score ^5^	6 [4, 7]	5 [4, 7]	6 [4, 7]	0.022 ^4^

^1^ n (%); Mean (SD); Median [IQR]; ^2^ Pearson’s chi-squared test; ^3^ Two-Sample *t*-test; ^4^ Wilcoxon rank-sum test; ^5^ FTCD—Fagerström Test of Cigarette Dependence.

**Table 3:** Tobacco dependence treatment outcomes.

	Total n = 2039 ^1^	Treatment Era		*p* Value ^2^
		Before COVID-19 n = 1221 ^1^	During COVID-19 n = 818 ^1^	
Treatment Outcome				0.440
Abstinence	839 (41%)	494 (40%)	345 (42%)	
Failure	1200 (59%)	727 (60%)	473 (58%)	

^1^ n (%); ^2^ Pearson’s chi-squared test.

**Table 4:** Number of visits per patient during the treatment period.

	Total n = 2039 ^1^	Treatment Era		*p* Value ^2^
		Before COVID-19 n = 1221 ^1^	During COVID-19 n = 818 ^1^	
All ^3^	6 [4, 8]	5 [3, 7]	7 [5, 9]	<0.001
Personal	4 [2, 6]	4 [2, 7]	4 [2, 6]	0.521
Telephone	1 [1, 2]	1 [0, 1]	2 [1, 3]	<0.001

^1^ Median [IQR]; ^2^ Wilcoxon rank-sum test; ^3^ Including failed to attend.

**Table 5:** The duration of tobacco dependence medication use.

	Total n = 2039 ^1^	Treatment Era		*p* Value ^2^
		Before COVID-19 n = 1221 ^1^	During COVID-19 n = 818 ^1^	
Number of days on medication	53 [15, 133]	51 [15, 133]	56 [18, 134]	0.699
Number of days without medication	346 [279, 379]	349 [286, 376]	341 [270, 385]	0.662

^1^ Median [IQR]; ^2^ Wilcoxon rank-sum test.

## Data Availability

The anonymized dataset analysed during the current study will be shared on reasonable request to the corresponding author.
